# Draft Genome Sequence of *Rhodococcus erythropolis* VSD3, a Diesel Fuel-Degrading and Plant Growth-Promoting Bacterium Isolated from *Hedera helix* Leaves

**DOI:** 10.1128/genomeA.01680-16

**Published:** 2017-02-23

**Authors:** Vincent Stevens, Sofie Thijs, Breanne McAmmond, Tori Langill, Jonathan Van Hamme, Nele Weyens, Jaco Vangronsveld

**Affiliations:** a Centre for Environmental Sciences, Hasselt University, Diepenbeek, Belgium; b Department of Biological Sciences, Thompson Rivers University, Kamloops, British Columbia, Canada

## Abstract

We report here the 6.55-Mb draft genome sequence of *Rhodococcus erythropolis* VSD3, a Gram-positive bacterium of the *Nocardiaceae* family, isolated from leaves of *Hedera helix* growing at a high-traffic city center in Belgium. The exploration of its genome will contribute to the assessment of its application as an inoculant in phylloremediation approaches.

## GENOME ANNOUNCEMENT

*Rhodococcus erythropolis* strains are reported in the context of plant growth promotion (1) and metabolization, including desulfurization, of diesel fuel (2–4). *R. erythropolis* VSD3 was isolated from the leaves of *Hedera helix* plants growing at a high-traffic city center in Belgium. *In vitro* analyses indicated that this bacterium utilizes diesel fuel as a carbon source and produces compounds related to plant growth promotion. Partial 16S rRNA gene sequence data revealed that VSD3’s closest relative is *Rhodococcus erythropolis* BG43 (GenBank accession no. CP011295).

RNA-free DNA was extracted from stationary-phase cells grown in LB medium using a PureLink genomic DNA minikit (Thermo Fisher Scientific, Waltham, MA, USA), prior to digesting and ligating sequencing adaptors/barcodes using an Ion Xpress Plus fragment library kit (Thermo Fisher Scientific). Processed DNA was size-selected (480 bp) on a 2% E-Gel SizeSelect agarose gel and purified using Agencourt AMPure XP beads (Beckman Coulter, Inc., Brea, CA, USA). The library dilution factor was determined using an Ion Universal library quantitation kit prior to amplification and enrichment with an Ion PGM Hi-Q Template OT2 400 kit on an Ion OneTouch 2 system. The enriched Ion Sphere Particles were quantified using an Ion Sphere quality control kit. Sequencing was performed on an Ion 316 Chip version 2 (Ion PGM system) with an Ion PGM Hi-Q View sequencing kit (Thermo Fisher Scientific).

In total, 2,234,103 reads (mean length, 263 bases) generated 588 Mb (552 Mb with ≥*Q*20) of data. Reads were assembled using SPAdes version 3.8.2 (5, 6) (uniform coverage mode; *k*-mers = 21, 33, 55, 77, 99, 127), trimmed into 38 contigs ≥1,000 bp, giving a consensus length of 6,549,507 bp at 84.2× coverage (largest contig, 1,761,316 bp; *N*_50_, 378,631 bp). The genome sequence of *R. erythropolis* BG43 was used as a reference to order the VSD3 contigs in Mauve (7, 8). Genome annotation was completed using RAST (9, 10) and NCBI’s PGAP (11). The genome of *R. erythropolis* VSD3 has a G+C content of 62.4% and includes 5,658 coding genes, 305 pseudogenes, eight rRNAs (5S, 16S, 23S), 52 tRNAs, and three noncoding RNAs (ncRNAs).

Genes connected with the degradation of *n*-alkanes were located in *R. erythropolis* VSD3’s genome, including homologues for all components of the *alkBFGHJKL* operon (12). *Pseudomonas putida* G7’s homocyclic aromatic hydrocarbon-degrading pathway (13) is also partly represented: homologues for all enzymes participating in the degradation of 2-hydroxymuconic semialdehyde to pyruvate and acetyl-coenzyme A (acetyl-CoA) are encoded in the genome. Concerning the degradation of heterocyclic aromatic hydrocarbons, genes homologous to the *dszABC* and *dszD* operon (14) are present, indicating that *R. erythropolis* VSD3 is capable of diesel fuel desulfurization. Further, genes related to plant growth-promoting characteristics were found: 1-aminocyclopropane-1-carboxylate deaminase activity and indole-3-acetic acid, acetoin, and siderophore production. *R. erythropolis* VSD3 is further being evaluated as an inoculant to enhance phylloremediation of environments contaminated with diesel fuel-associated air pollutants.

### Accession number(s).

This whole-genome sequencing project has been deposited in GenBank under the accession no. MLKO00000000. The version described in this paper is the first version, MLKO00000000.1
